# In this month’s Bulletin

**DOI:** 10.2471/BLT.22.001022

**Published:** 2022-10-01

In editorials this month, Kapil Narain et al. (582) discuss strategies for malaria vaccination during the COVID-19 pandemic in African countries. Dévora Kestel et al. (583) present a WHO report on the transformation needed in mental health care.

Vijay Shankar Balakrishnan and Gary Humphreys (586–587) report on the ways in which the monkeypox epidemic is highlighting the risks entailed in neglecting local outbreaks. Vera Cordeiro talks to Andréia Azevedo Soares (588–589) about creating a community-level strategy to tackle the interlinked conditions of poverty and disease.

## Bangladesh, India, Indonesia, Malaysia, Viet Nam

### Providing diagnosis and treatment for hypertension 

Pascal Geldsetzer et al. (601–609) analyse survey data from demographic surveillance sites.

## Bangladesh, Indonesia, Pakistan

### Upgrading routine health information systems

Tigest Tamrat et al. (590–600) transform paper registers into a digital system.

## Brazil 

### Screening for diabetic retinopathy

Fernando Korn Malerbi & Gustavo Barreto Melo (643–647) test the feasibility of using artificial intelligence.

## India 

### How are antibiotics being used?

Aashna Mehta et al. (610–619) track sales data.

## Sao Tome and Principe 

### Reducing harms from alcohol

Zila M Sanchez et al. (628–635) describe the process of developing legislation.

## WHO African Region

### Development, production and regulation of COVID-19 vaccines

Geofrey Makenga et al. (651–652) make the case for regional capacity development.

## Global

### Tobacco product waste

Juleen Lam et al. (620–627) model global economic costs. 

### Accessibility of children’s medicines

Iris R Joosse et al. (636–642) delineate data gaps. 

### Inequalities in COVID-19 mortality

Joseph Friedman et al. (648–650) define a research agenda.

